# Diseases of the Heart and Arterial System

**Published:** 1904-03

**Authors:** 


Diseases of the Heart and Arterial System. Designed to be a practical presentation of the subject for the use of students and practitioners of medicine. By Robert H. Babcock, A. M., M. D., Professor of Clinical Medicine and Diseases of the Chest, University of Chicago, etc. With three colored plates and 139 illustrations. Pages, 837. Price, cloth, $5.00. New York: D. Appleton & Co. 1903.

This work can be considered a treatise, though the author does not say so in his preface. At first glance the volume looks large for the subject, but if any close observer will study carefully the article on any of the diseases treated, pericarditis for instance, he will conclude, as did the reviewer, that the author is master of the subject; that he has not detailed too extensively, but, on the contrary, has put forth a clear, practical article. The othor chapters of the book are as well written as the one on pericarditis. Taking it altogether, Babcock has written a book that will at once take its place as a standard on diseases of the heart and arterial system. T. J. B.

